# Novel Basophil- or Eosinophil-Depleted Mouse Models for Functional Analyses of Allergic Inflammation

**DOI:** 10.1371/journal.pone.0060958

**Published:** 2013-04-08

**Authors:** Kunie Matsuoka, Hiroshi Shitara, Choji Taya, Kenji Kohno, Yoshiaki Kikkawa, Hiromichi Yonekawa

**Affiliations:** 1 Mammalian Genetics Project, Tokyo Metropolitan Institute of Medical Science, Tokyo, Japan; 2 Laboratory for Transgenic Technology, Tokyo Metropolitan Institute of Medical Science, Tokyo, Japan; 3 Graduate School of Biological Sciences, Nara Institute of Science and Technology, Nara, Japan; Louisiana State University Health Sciences Center, United States of America

## Abstract

Basophils and eosinophils play important roles in various host defense mechanisms but also act as harmful effectors in allergic disorders. We generated novel basophil- and eosinophil-depletion mouse models by introducing the human diphtheria toxin (DT) receptor gene under the control of the mouse CD203c and the eosinophil peroxidase promoter, respectively, to study the critical roles of these cells in the immunological response. These mice exhibited selective depletion of the target cells upon DT administration. In the basophil-depletion model, DT administration attenuated a drop in body temperature in IgG-mediated systemic anaphylaxis in a dose-dependent manner and almost completely abolished the development of ear swelling in IgE-mediated chronic allergic inflammation (IgE-CAI), a typical skin swelling reaction with massive eosinophil infiltration. In contrast, in the eosinophil-depletion model, DT administration ameliorated the ear swelling in IgE-CAI whether DT was administered before, simultaneously, or after, antigen challenge, with significantly lower numbers of eosinophils infiltrating into the swelling site. These results confirm that basophils and eosinophils act as the initiator and the effector, respectively, in IgE-CAI. In addition, antibody array analysis suggested that eotaxin-2 is a principal chemokine that attracts proinflammatory cells, leading to chronic allergic inflammation. Thus, the two mouse models established in this study are potentially useful and powerful tools for studying the *in vivo* roles of basophils and eosinophils. The combination of basophil- and eosinophil-depletion mouse models provides a new approach to understanding the complicated mechanism of allergic inflammation in conditions such as atopic dermatitis and asthma.

## Introduction

IgE, mast cells, basophils, and eosinophils are important elements in allergic inflammation. Mast cells and basophils have long been considered primary effector cells in allergic disorders such as asthma, hay fever, and anaphylaxis. Allergen-specific IgE, synthesized in response to allergens in the environment, binds to FcεRI on the surface of mast cells and basophils. Cross-linking of receptor-bound IgE molecules upon re-exposure to specific allergens results in the release of chemical mediators, such as histamine and leukotriene C4, that produce the allergic response [1], [2], [3], [4], [5]. Principal among the cells drawn to sites of mediator release is the eosinophil. The effector functions of eosinophils appear to be derived primarily from the release of lipid mediators and proteins, including cytokines and granule proteins. Eosinophil degranulation results in the release of several cytotoxic cationic granule proteins [6]. Cytotoxic eosinophils are harmful to foreign invaders within the body and can also become detrimental to the host organs through an intricate immunological pathway [7].

Many studies have demonstrated that mast cells are key effector cells in IgE-associated immune responses, including allergic disorders and certain protective immune responses to parasites [8], [9], [10], [11], [12], [13]. *In vivo* studies using mast cell-deficient mouse strains carrying mutations in the *Kit* or *stem cell factor (SCF)* gene, such as WBB6F1-*W/W^v^*, C57BL/6-*W^sh^/W^sh^*, and WBB6F1-*Sl^d^/Sl^d^* [14], [15], [16] have supplied consequential evidence of the essential role of mast cells in both physiological and pathological environments. Mast cells have been shown to contribute to a complex range of immune functions that go far beyond allergies and include the development of autoimmune disorders and peripheral tolerance and the initiation and maintenance of adoptive and innate host responses [17]. Two independent mouse lines with eosinophil deficiency have been developed. One group created a transgenic (Tg) mouse line, designated PHIL, using an eosinophil-specific promoter from the gene for eosinophil peroxidase (EPO) to drive the expression of a cytocidal protein diphtheria toxin A chain [18]. PHIL mice are specifically devoid of eosinophils and are protected from the development of airway hyperresponsiveness in an experimental asthma model. Another group developed an eosinophil lineage-ablated mouse line (Δdbl GATA) by depletion of the high-affinity GATA-binding site in the GATA-1 promoter [19]. Δdbl GATA mice are significantly protected from peribronchiolar collagen deposition and increases in airway smooth muscle mass [20].

In contrast to the extensive studies on mast cells and eosinophils, the *in vivo* roles of basophils have been poorly studied and defined. We previously demonstrated that basophils are responsible for the development of IgE-mediated chronic allergic inflammation (IgE-CAI) independently of T cells and mast cells [21]. A single subcutaneous challenge of multivalent allergens elicited not only immediate- and late-phase ear swelling but also delayed-onset ear swelling with massive eosinophil infiltration in mice that had been passively sensitized with antigen-specific IgE. We found that basophils were essential for the development of IgE-CAI [21]. However, a roadblock to studying basophil functions *in vivo* is the lack of appropriate animal models such as basophil-deficient mice.

In long expectation, an mAb specific to mouse basophils was generated. The mAb, named Ba103 and specific to CD200R3, depletes 80–90% of the basophils from the mouse peripheral blood and the spleen following i.v. injection [22], [23]. Ba103 treatment of mice completely abolished the development of IgE-CAI and greatly suppressed penicillin V-induced IgG1-mediated anaphylaxis [24]. However, the phenotype of these antibody-treated mice may be ascribed to basophil depletion, to deleterious effects on mast cells, or to both [25]. Recently, 2 kinds of basophil ablation mouse models were generated. One is the Tg mouse *Mcpt8^DTR^*, expressing the human diphtheria toxin (DT) receptor (hDTR) [26], and the other is the Tg mouse *Mcpt8Cre*, which expresses the Cre recombinase [27] under the control of the regulatory elements for mast cell protease 8 (*Mcpt8*). *Mcpt8^DTR^* mice constitute a DT-induced basophil ablation model and *Mcpt8Cre* mice are constitutively deficient for basophils. DT administration to *Mcpt8^DTR^* mice led to the transient depletion of basophils from the bone marrow, peripheral blood, and spleen [26]. In *Mcpt8Cre* mice, more than 90% of basophils were spontaneously deleted by Cre toxicity resulting from nonspecific recombination events of cryptic loxP sites in the mouse genome [27]. Moreover, an hDTR Tg mouse controlled by the *IL-4* promoter, its 5′ enhancer, and the proximal 3′ untranslated region was recently generated as a basophil ablation mouse model [28].

We have now newly established mouse models lacking basophils and eosinophils to study critical roles in immunological responses. These Tg mice express the hDTR under control of the mouse *CD203c* or *EPO* promoter in the C57BL/6 genetic background. These mouse models exhibited selective and efficient depletion of target cells upon DT administration.

## Materials and Methods

### Antibodies

Goat polyclonal antibody specific for human heparin-binding EGF-like growth factor (HB-EGF, also known as hDTR) was purchased from GT (Minneapolis, MN, USA), and rabbit polyclonal antibody for mouse CCR3 was purchased from ProSci (Poway, CA, USA). For flow cytometric analyses, FITC-conjugated mAbs for mouse IgE (R35-72), and CCR3 (83101) and PE-conjugated mAbs specific for CD49b (DX5), c-kit (2B8), and Siglec-F (E50-2440) were purchased from BD PharMingen (San Diego, CA, USA). Alexa Fluor 488-conjugated anti-goat IgG and Alexa Fluor 594 anti-rat and anti-rabbit IgG were purchased from Invitrogen (Eugene, OR, USA).

### Construction of Transgenes

DNA fragments corresponding to the promoter regions of *CD203c* and *EPO* were amplified by PCR from BALB/c mouse genomic DNA by using the following primer sets: 5′-ATA AGA ATG CGG CCG CCT AGC CGA TGT CCT TTT CC-3′ and 5′-CGG GAT CCG TAG CTG TGT AAA CCT GTC-3′ for *CD203c* and 5′-ATA AGA ATG CGG CCG CTT CAG GGC TTG CGA CTT GTG T-3′ and 5′-TCG GAT CCA GAT GCC ACG GAG GTG AGT GTA C-3′ for *EPO*. The *albumin* promoter in the plasmid pMS7, which is the transgene construct of the conditional hepatocyte ablation mouse model, was replaced at the *Not*I-*Bam*HI site by either the 6.4-kbp *CD203c* or the 6.0-kbp *EPO* promoters [29]. The 8.8- or 8.4-kbp *Not*I-*Xho*I fragment, containing the *CD203c* or *EPO* promoter, *β-globin* intron, *hDTR* cDNA, and the polyadenylation signals of *β-globin* and SV40, was excised and purified using the QIAquick gel extraction kit (QIAGEN, Valencia, CA, USA) and the Wizard DNA Clean-Up System (Promega, Madison, WI, USA).

### Generation of Tg Mice

Tg mice were generated by microinjection of the DNA constructs into pronucleus stage oocytes from C57BL/6 mice. Genomic DNA was purified from tail biopsies using the Wizard Genomic DNA Purification Kit (Promega). PCR genotyping was performed for genomic DNA using the primer set: 5′-CAA CTA CAT CCT GGT CAT CAT C-3′ and 5′-CAG ACA GAT GAC AGC ACC ACA G-3′ for the junction between the β-globin intron and hDTR cDNA. This study was carried out in strict accordance with the recommendations outlined in the Guidelines for Proper Conduct of Animal Experiments by the Science Council of Japan. The protocol was approved by the Institutional Animal Experiment Committee of Tokyo Metropolitan Institute of Medical Science (permit number, 11-059). All surgery was performed under isoflurane anesthesia, and all efforts were made to minimize suffering.

### RT-PCR

Basophils were purified from the peripheral blood of mice by positive selection using the MACS system (Miltenyi Biotech, Bergisch Gladbach, Germany) with FITC-conjugated anti-IgE mAb and anti-FITC microbeads (Miltenyi Biotech). The basophil-specific expression of hDTR mRNA was analyzed by RT-PCR. PCR was performed on cDNA from IgE-positive or IgE-negative peripheral leukocytes using the following primer sets: 5′-TTA TCC TCC AAG CCA CAA GCA CTG-3′ and 5′-AGA CAG ACA GAT GAC AGC ACC ACA G-3′ for hDTR and 5′-TCT GGC TCC TAG CAC CAT GAA GAT C-3′ and 5′-TCA GTA ACA GTC CGC CTA GAA GCA C-3′ for β-actin.

### Administration of DT

DT was purified from the conditioned medium of the *C. diphtheria* strain PW8 as described previously [32]. The mice were i.p. administered various doses of DT (5–50 µg/kg body weight) or PBS alone as the control.

### Flow Cytometry, Cytospin Preparation, and Histological Analysis

Peripheral blood cells, spleen cells, and peritoneal cells were depleted of RBCs by lysis with hypotonic buffer. For flow cytometric analysis, cells were preincubated with anti-FcγRII/III (2.4G2) mAb (BD PharMingen) and normal rat serum on ice for 30 min to prevent the nonspecific binding of other Abs. The remaining cells were stained with the indicated combinations of Abs following 7-aminoactinomycin (7-AAD) incubation and analyzed using FACSCalibur (BD Biosciences, San Jose, CA, USA). Fluorescence data were gated on forward scatter (FSC) vs. 7-AAD fluorescence dual-parameter contour plots for excluding debris and clumps. For cytospin slides of peripheral leukocytes, cell smears prepared using Cytospin3 (Thermo Fisher Scientific, Waltham, MA, USA) were fixed in 95% EtOH for 30 min at room temperature (RT), stained with the indicated combinations of Abs, and analyzed with a LSM 520 META microscope (Carl Zeiss, Göttingen, Germany). For histological examination under a microscope, ear specimens were fixed with 10% formalin and embedded in paraffin; subsequently, 5-µm-thick slices were obtained. The sections were stained with an anti-CCR3 polyclonal antibody using the R.T.U. VECTASTAIN Universal Quick Kit (Vector Laboratories, Burlingame, CA, USA) following hematoxylin staining.

### IgE-mediated Chronic Allergic Skin Inflammation

IgE-mediated chronic allergic skin inflammation (IgE-CAI) was elicited as described earlier [21]. In brief, mice were passively sensitized with 300 µg trinitrophenyl (TNP)-specific IgE mAb (IGELb4) by i.v. injection. One day later, 10 µg TNP-conjugated ovalbumin (TNP_11_-OVA; Bioresearch Technologies, Novato, CA, USA) in 10 µl PBS was injected subcutaneously into the right ear of each mouse, and an equal amount of OVA was injected into the left ear. Ear thickness was measured with a PEACOCK G-1A dial thickness gauge (Ozaki MFG, Tokyo, Japan) at the indicated time points.

### Induction of Systemic Anaphylaxis

Mice were given an i.p. injection of DT (25 or 50 µg/kg body weight). In order to induce passive anaphylaxis, they were sensitized with 300 µg TNP-specific IgE mAb (IGELb4) 3 days later by i.v. injection in 200 µl PBS. One day later, i.v. challenge with 50 µg TNP_13_-OVA in 200 µl in PBSwas performed. In order to induce anti-FcγRII/III antibody-elicited anaphylaxis, systemic anaphylaxis was induced by i.v. injection of 500 µg of rat anti-FcγRII/III (2.4G2) antibodies in 200 µl PBS 4 days later. The rectal temperature of each mouse was measured using a digital thermometer (Shibaura Electronics, Saitama, Japan).

### Semiquantitative Analysis of Cytokines and Chemokines Found in Ear Skin on IgE-CAI

IgE-CAI was elicited as mentioned above. Mice ears were excised and homogenized in PBS at 4 days after antigen injection. The major cytokines and chemokines in 200 µg of extracted total proteins were analyzed semiquantitatively by using antibody arrays for 97 cytokines (Mouse Cytokine Antibody Array G Series 6; RayBiotech, Norcross, GA, USA) following the manufacturer’s protocol.

## Results

### Generation of Basophil-depleted Mice for Selective and Inducible Ablation of Basophils

**Figure 1:**
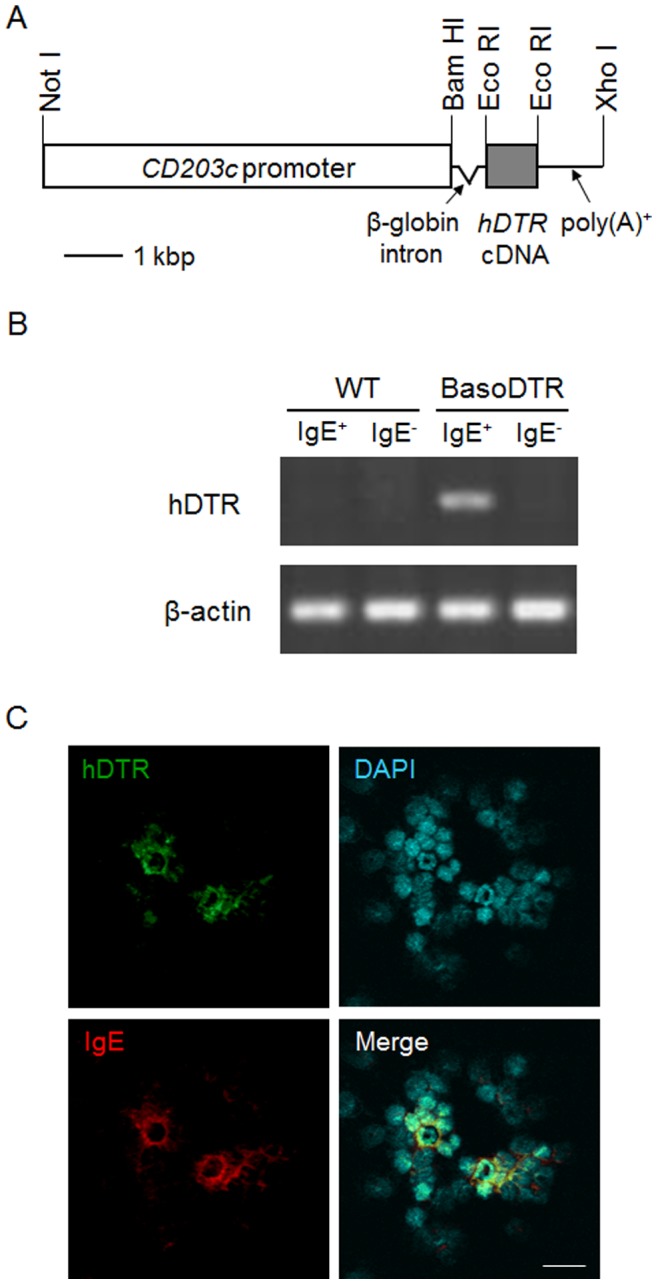
Generation of BasoDTR mice for the selective and inducible ablation of basophils. (A) Construct of a transgene for BasoDTR mice. The *alb* promoter of the original TRECK cassette was replaced with the *CD203c* promoter to allow the exclusive expression of hDTR on basophils [29]. (B) Basophils were purified as IgE-positive cells from the peripheral blood of BasoDTR and WT mice by using the MACS system. RT-PCR analysis was performed to examine the specific expression of hDTR mRNA in basophils. (C) Cytospin slides of the peripheral leukocytes of BasoDTR mice were stained with antibodies against hDTR (green), IgE (red), and DAPI (nuclei, blue). Scale bar = 20 µm.

To develop a system in which basophil function could be studied *in vivo*, we used our toxin receptor-mediated conditional cell knockout (TRECK) method, a conditional ablation system mediated by the hDTR [29]. Because *CD203c* has been identified as a basophil activation marker [30], [31] and because recent studies have revealed that the expression of CD203c on basophils is useful in the diagnosis of food allergies and asthma [31], [32], the *CD203c* promoter was introduced in place of the *alb* promoter of the original TRECK cassette to facilitate the exclusive expression of hDTR on basophils (Fig. 1A). The *CD203c*–containing TRECK construct was injected into C57BL/6 fertilized eggs to generate Tg mice. Basophils were prepared from peripheral blood by positive selection using the MACS system with an IgE-specific antibody, as mentioned above, and RT-PCR analysis was performed to examine whether specific expression of hDTR mRNA occurred on basophils. As expected, hDTR mRNA was expressed only in IgE-positive cells of basophil-depleted (BasoDTR) mice; no expression signals were detected in the IgE-negative cell fraction of BasoDTR mice or in either fraction of wild-type (WT) mice (Fig. 1B). Moreover, immunostaining of peripheral blood leukocytes prepared by cytospin revealed that hDTR was detected on the surface of basophils stained with an IgE-specific antibody (Fig. 1C). These results suggest that hDTR was expressed specifically on basophils in the BasoDTR mice.

**Figure 2:**
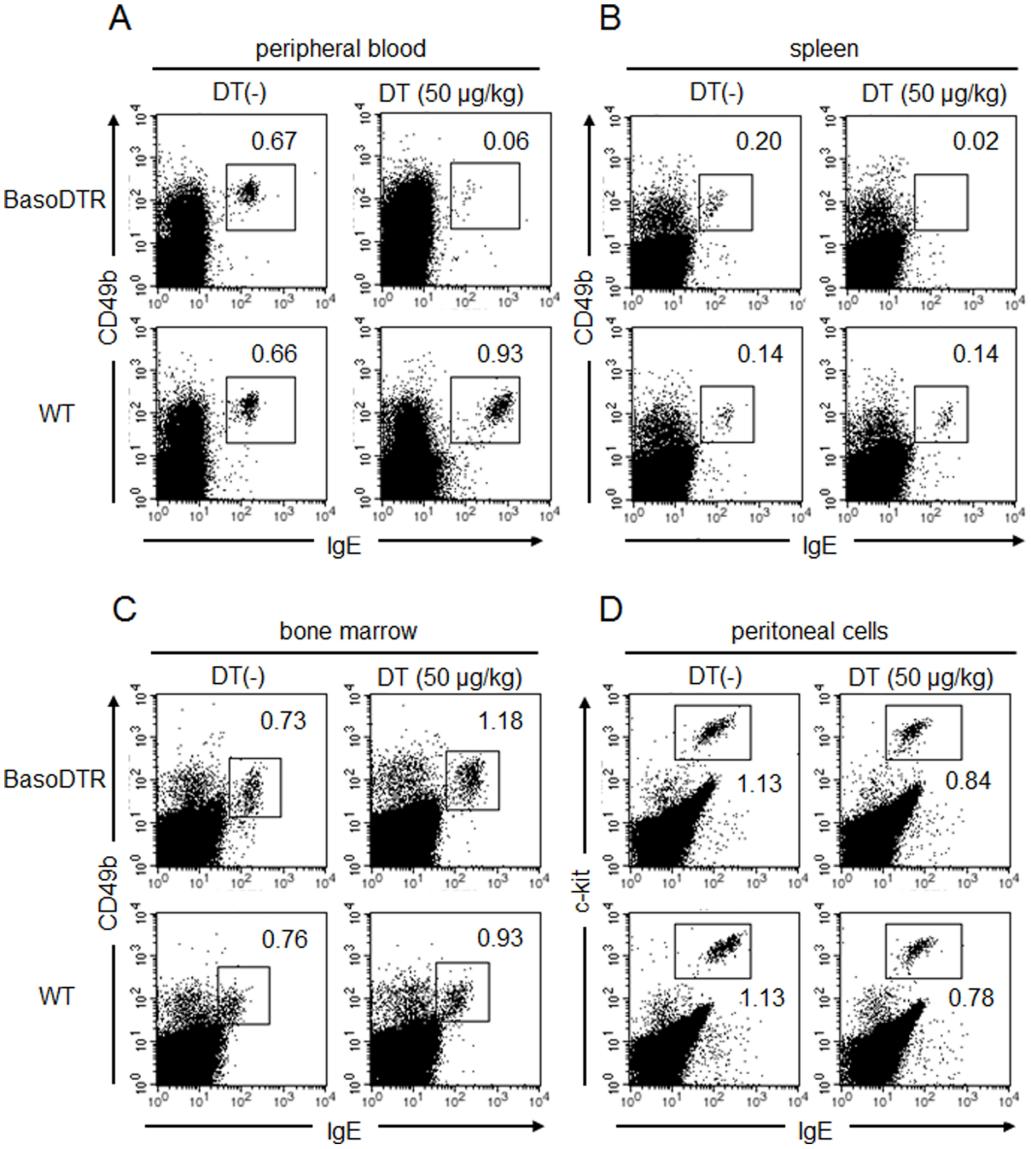
Basophil-specific ablation after DT administration in BasoDTR mice. BasoDTR and WT mice were treated once i.p. with DT (50 µg/kg body weight). After 4 days, basophils in the peripheral blood (A), spleen (B), bone marrow (C), and peritoneal mast cells (D) were analyzed by flow cytometry. The percentages of basophils (IgE^+^CD49b^+^) and mast cells (IgE^+^c-kit^+^) are indicated.

To assess whether administration of DT leads to selective depletion of basophils *in vivo*, 50 µg/kg of DT was injected into 8-week-old BasoDTR mice and WT mice. On the 4th day after injection, the basophil population was subjected to flow cytometric analysis. As expected, drastic decreases in the basophil fraction were observed in both the peripheral blood and the spleen in BasoDTR mice, whereas no decrease in the basophil fraction was detected in WT mice (Fig. 2A and B). However, DT administration did not affect other cell types, including peritoneal mast cells, peripheral blood lymphocytes, and granulocytes (Fig. 2D and Table 1). Similar phenomena were also observed in the spleen (data not shown). These results clearly demonstrated that basophil-specific depletion occurred in the peripheral blood and spleen of BasoDTR mice. In contrast, no decrease was observed in the basophil population in the bone marrow (Fig. 2C).

**Table 1:** Percentage of leukocytes in peripheral blood and mast cells in peritoneal exudate cells.

cell type	WT	BasoDTR
basophils	1.05±0.20	0.17±0.13
eosinophils	1.38±0.34	1.35±0.57
neutrophils	4.19±1.22	4.76±1.94
T cells	35.85±4.01	26.80±3.96
B cells	45.44±2.10	50.20±7.20
mast cells	0.70±0.26	0.70±0.02

Three WT and BasoDTR mice were i.p. administered 50 µg/kg DT. After 4 days, FACS analysis of peripheral blood cells and peritoneal exudate cells was performed. The percentages of the indicated cells from the total cells have been shown as mean ± SD values.

We performed RT-PCR analysis to detect the expression of CD203c and hDTR in BasoDTR mice (Fig. S1A). CD203c mRNA expression was detected in bone marrow basophils but not in splenic basophils. In contrast, hDTR mRNA was expressed in both types of basophils. Therefore, we tried to detect hDTR proteins in the bone marrow basophils by using western blotting and cytometric analyses. However, these methods were unsuccessful (data not shown). We identified two possible reasons for our failure. First, the purification of basophils from bone marrow cells is necessary to detect hDTR proteins with western blotting. However, the population of basophils in the bone marrow is too small to achieve adequate purification of the cells at the detection level of western blotting. Second, for the cytometric analysis, we used two commercially available monoclonal antibodies and two polyclonal antibodies against hDTR. However, we found no anti-hDTR antibodies with a high enough binding capacity for hDTR proteins in both bone marrow basophils and splenic basophils to detect hDTR proteins. Therefore, the question remains whether bone marrow basophils of BasoDTR mice produce hDTR proteins. We are currently investigating this question by generating new monoclonal or polyclonal antibodies.

**Figure 3:**
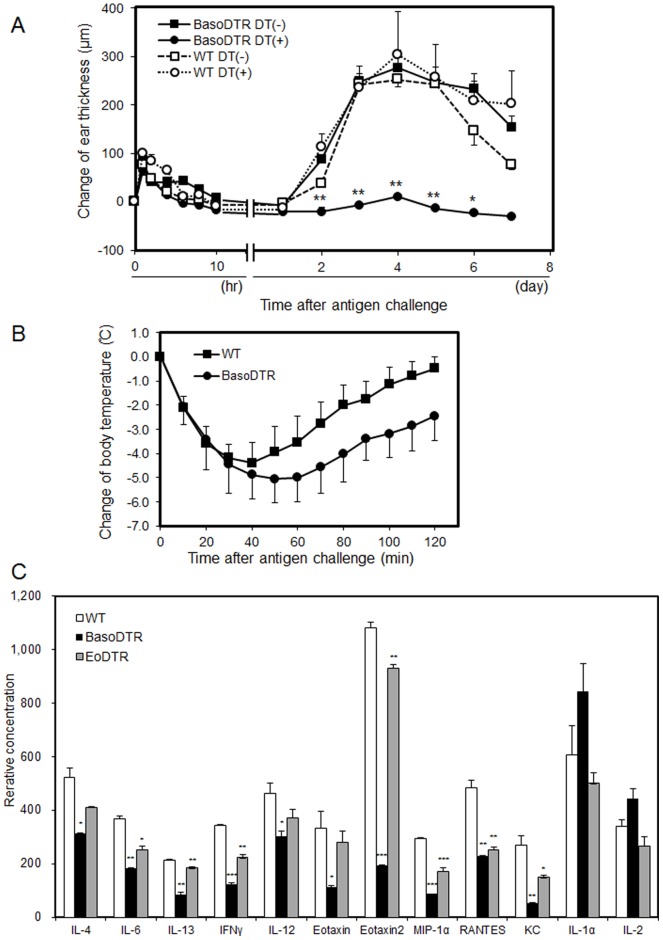
IgE-CAI and the expression of cytokines and chemokines in antigen-challenged ears. (A) BasoDTR (n = 4) and WT mice (n = 4) were treated once i.p. with 25 µg/kg DT or with PBS 2 days before and were injected i.v. with 300 µg TNP-specific IgE 1 day before challenge with 10 µg TNP-OVA. Ear thickness was measured at the indicated time points. The kinetics of changes in ear thickness after antigen challenge are shown. Data have been expressed as mean ± SEM and are representative of 4 repeated experiments. *P* values of DT-administered BasoDTR mice versus WT mice are indicated. *p<0.05, **p<0.01. (B) WT and BasoDTR mice were passively sensitized with i.v. injections of 200 µl diluted ascites containing 300 µg of TNP-specific IgE at 1 day before 50 µg of TNP_13_-OVA was administered. The change in rectal temperature over time after allergen challenge is shown. Data have been expressed as mean ± SEM and are representative of 4 repeated experiments. (C) The ears of WT (n = 4), BasoDTR (n = 4), and EoDTR (n = 4) mice were excised and homogenized in PBS at 4 days after antigen challenge. The major cytokines and chemokines in 200 µg of extracted total proteins were analyzed semiquantitatively by using antibody arrays. Relative concentrations have been indicated in terms of mean ± SEM. The *p* values of BasoDTR mice versus WT mice and of EoDTR mice versus WT mice are indicated. *p<0.05, **p<0.01. Statistical analysis was performed using the Student’s *t* test.

CD203c, the promoter used in this study, exists on basophils and, in some cases, on mast cells in humans [33]. RT-PCR analysis detected neither CD203c nor hDTR mRNA expression in the mast cells of peritoneal exudate cells of BasoDTR mice (Fig. S1A). To clarify the functional influence of mast cell depletion after DT administration, we subjected BasoDTR mice to test the induction of IgE-mediated systemic anaphylaxis which is caused by mast cells [34], [24]. WT and BasoDTR mice were administered 50 µg/kg of DT 4 days before treatment, passively sensitized with TNP-specific IgE, and challenged with i.v. injection of TNP_13_-OVA. Both WT and BasoDTR mice developed systemic anaphylaxis, as determined by a drastic drop in body temperature, although the decrease in temperature in BasoDTR mice was notably greater than that in WT mice (Fig. 3B) mice. Therefore, mast cells clearly remained functional after DT administration.

### DT Administration Eliminates Basophil Functions *in vivo*

IgE-CAI is the delayed-onset ear swelling that typically starts on day 1 or 2 after antigen challenge and peaks on day 3 or 4 [23]. Recent studies have suggested that basophils may function as initiators as well as, or rather than, effectors and contribute to the recruitment of other proinflammatory cells such as eosinophils and neutrophils [23], [25]. BasoDTR mice were given 25 µg/kg of DT 2 days before and a TNP-specific IgE was i.v. injected 1 day before antigen challenge. Treatment with DT almost completely abolished the development of IgE-CAI in BasoDTR mice (Fig. 3A). This indicates that the effects of basophils in the induction of IgE-CAI were eliminated by DT administration. However, mast cells were functional since the immediate-phase ear swelling appeared within 1 hour after antigen challenge [21]. These results are consistent with those of the flow cytometric analysis described above (Fig. 2D). Cytokines and chemokines present in the ears 4 days after antigen injection were measured semiquantitatively by using antibody arrays. Fig. 3B shows the relative concentration of each cytokine and chemokine. The concentrations of Th2 cytokines (IL-4, IL-6, and IL-13), as well as Th1 cytokines (IFNγ and IL-12) and several chemokines, had decreased in BasoDTR mice. In contrast, the concentrations of IL-1α and IL-2 had slightly increased in BasoDTR mice. Eotaxin-2, in particular, was expressed at a higher level than other chemokines in WT and in eosinophil-depleted (EoDTR) mice; however, its expression was particularly diminished in BasoDTR mice.

**Figure 4:**
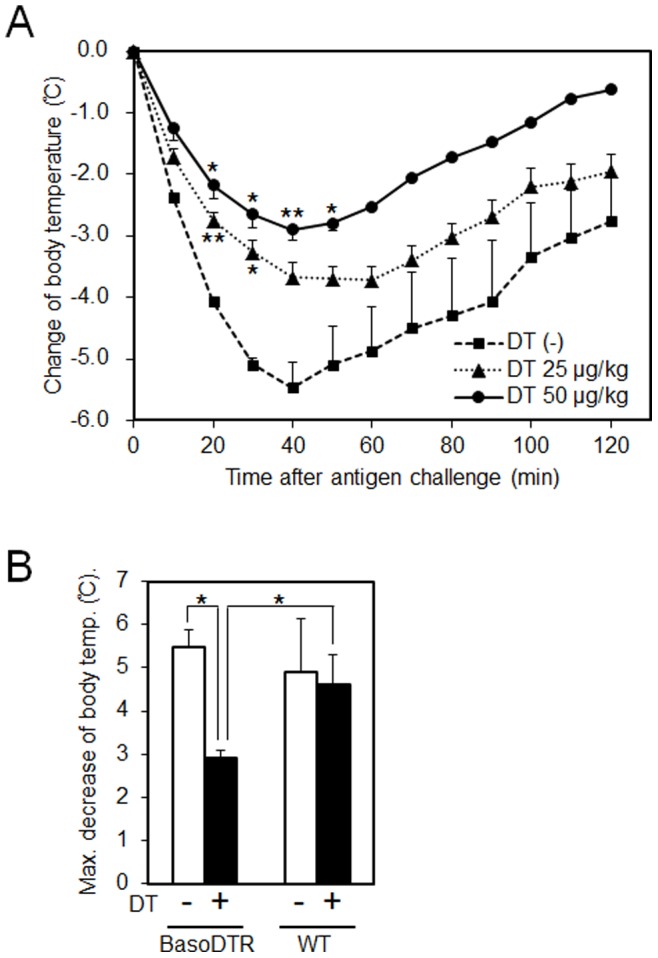
IgG-mediated systemic anaphylaxis decreased in BasoDTR mice. (A) BasoDTR mice (n = 4 per group) were treated once i.p. with 25 or 50 µg/kg of DT or PBS at 4 days prior to and were i.v. injected with 500 µg rat anti-FcγRII/III (2.4G2) antibodies in 200 µl PBS. Body temperature was measured at the indicated time points. Data have been expressed as mean ± SEM and are representative of 4 repeated experiments. The *p* values of DT-administered mice versus control mice are indicated. *p<0.05, **p<0.01. (B) The maximum decrease in the body temperature of BasoDTR and WT mice (n = 4 per group) with or without DT treatment were compared. Data have been expressed in terms of mean ± SEM and are representative of 4 repeated experiments. *p<0.05. Statistical analysis was performed using the Student’s *t* test.

Basophils play a pivotal role in IgG-mediated systemic anaphylaxis in mice [24]. We examined whether DT treatment attenuates the drop in body temperature in BasoDTR mice in systemic anaphylaxis elicited by i.v. injection of the mAb against FcγRII/III (2.4G2). Forty minutes after the injection of 500 µg 2.4G2, BasoDTR mice without DT treatment exhibited a significant drop in body temperature (Fig. 4A). The maximal decrease in body temperature was identical to that seen in WT mice (Fig. 4B). We also compared temperature responses in BasoDTR mice that had been given 25 µg or 50 µg of DT. DT treatment attenuated the drop in body temperature in a dose-dependent manner (Fig. 4A). None of the mice died because of the antibody injected during any of these experiments.

### Generation of EoDTR Mice for Selective and Inducible Ablation of Eosinophils

**Figure 5:**
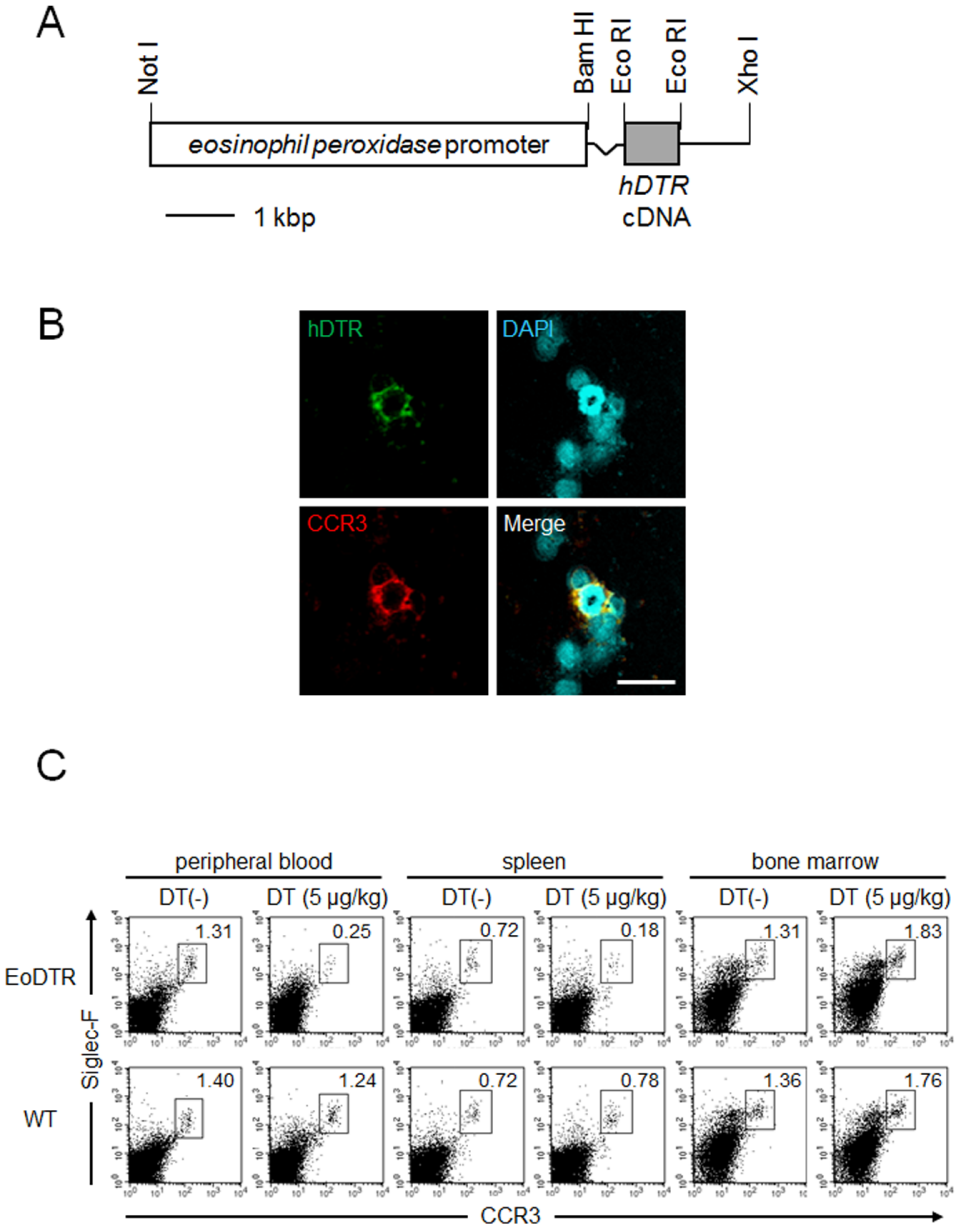
Generation of EoDTR mice for the selective and inducible ablation of eosinophils. (A) The *alb* promoter of the original TRECK cassette was replaced with the *EPO* promoter to identify the exclusive expression of hDTR on eosinophils [29]. (B) Cytospin slides of the peripheral leukocytes of EoDTR mice were stained with antibodies against hDTR (green), CCR3 (red), and DAPI (nuclei, blue). Scale bar = 20 µm. (C) EoDTR and WT mice were treated once i.p. with DT (5 µg/kg body weight). After 3 days, eosinophils in the peripheral blood, spleen, and bone marrow were analyzed by flow cytometry. The percentage of eosinophils (CCR3^+^Siglec-F^+^) is indicated.

Because little is known about the mechanism of cooperative function between basophils and eosinophils in IgE-CAI, we tried to follow that line of research. We generated EoDTR mice that express hDTR in eosinophils to induce eosinophil depletion specifically via treatment with DT. hDTR expression was driven by the murine *EPO* promoter. EPO is one of the most abundant granule cationic proteins in eosinophils. The recombinant gene for transgenesis was constructed via simple replacement of the *CD203c* promoter with the *EPO* promoter, as demonstrated in Fig. 5A. The recombinant gene was injected into C57BL/6 fertilized eggs to generate Tg mice for specific eosinophil ablation. Peripheral blood leukocyte preparations from EoDTR mice were subjected to a cytospin protocol followed by immunostaining with an anti-hDTR polyclonal antibody.

We found that hDTR was expressed exclusively on the surface of CCR3-expressing eosinophils (Fig. 5B). Flow cytometric analysis revealed a remarkable decrease in eosinophils in the peripheral blood and spleens of EoDTR mice 3 days after DT administration at a dose of 5 µg/kg (Fig. 5C). In contrast, a moderate increase in eosinophils was detected in the bone marrow of EoDTR as well as WT mice. We performed RT-PCR analysis to detect the expression of EPO and hDTR (Fig. S1B). EPO mRNA expression was detected in bone marrow eosinophils but not in splenic eosinophils. However, hDTR mRNA was expressed in both types of eosinophils. The cell population of eosinophils was double that of basophils. Therefore, neither the western blot nor the flow cytometric analysis detected hDTR signals on eosinophils, possibly owing to effects on the same factors described above for basophils. One possibility is that the expression of EPO and hDTR mRNA and hDTR protein do not correspond quantitatively to each other. DT administration in EoDTR mice did not decrease the levels of other cell types, including lymphocytes and granulocytes (Table 2). These results indicate the feasibility of specific and substantial depletion of eosinophils *in vivo* in EoDTR mice through DT administration.

**Table 2:** Percentage of leukocytes in peripheral blood after DT administration.

cell type	WT	EoDTR
eosinophils	1.43±0.49	0.29±0.06
neutrophils	11.44±1.95	19.97±8.7
basophils	0.96±0.24	0.84±0.04
T cells	20.63±2.39	20.94±2.39
B cells	51.41±2.88	45.64±5.53

Three WT and EoDTR mice were i.p. administered 5 µg/kg DT. After 3 d, FACS analysis of peripheral blood cells was performed. The percentages of the indicated cells from the total cells have been shown as mean ± SD values.

### DT Administration into EoDTR Mice Induces a Drastic Reduction in Ear Swelling in IgE-CAI

**Figure 6:**
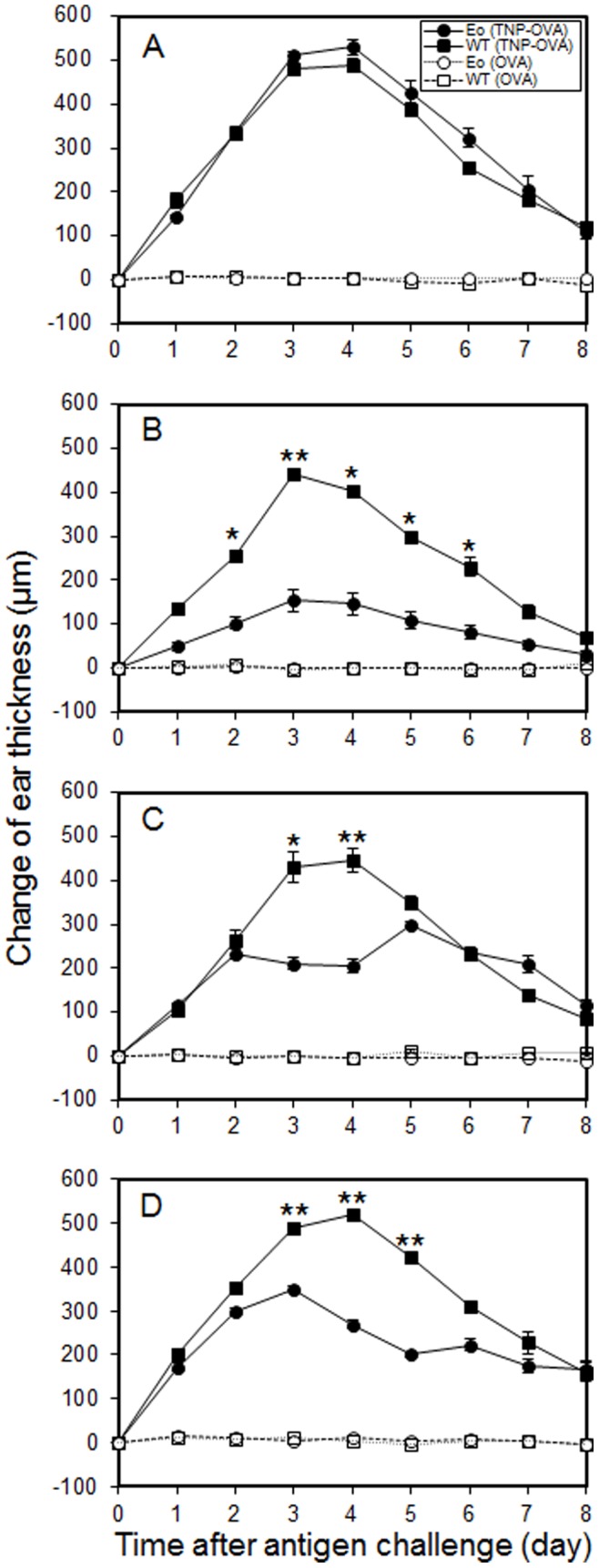
IgE-CAI in EoDTR mice. Ear swelling in IgE-CAI in mice receiving PBS (A, n = 5) or 5 µg/kg of DT at 2 days before (B, n = 5), at the same time as (C, n = 7), or 2 days after (D, n = 6) the antigen challenge were measured at the indicated time points. Data have been expressed in terms of mean ± SEM and are representative of 2 repeated experiments. The *p* values of DT-treated EoDTR mice versus DT-treated WT mice are indicated. *p<0.05, **p<0.01. Statistical analysis was performed using the Student’s *t* test.

In the chronic phase of IgE-CAI, the swelling of ear skin is the most definitive indicator of the massive infiltration of proinflammatory cells, including eosinophils and neutrophils [21], [32]. EoDTR mice without DT administration showed delayed-onset ear swelling comparable to that of WT mice (Fig. 6A). We investigated ear swelling in IgE-CAI in mice receiving 5 µg/kg of DT 2 days before, at the same time, or 2 days after antigen challenge. Treatment with DT partially ameliorated the ear swelling, regardless of the timing of DT administration (Fig. 6B, C and D). When mice were treated with DT at 2 days before the antigen challenge, the ear thickness was less than 50% of that seen in WT mice throughout the entire period of observation (Fig. 6B). When DT was administered at the same time as antigen challenge, ear swelling was attenuated to the same level as that shown in Fig. 6B on days 3 and 4 (Fig. 6C). DT treatment on day 2 resulted in reduction of ear swelling from day 3 to day 6 (Fig. 6D). In contrast, WT mice showed no change in ear swelling at any time during the treatment period. The levels of cytokines and chemokines present in the ears undergoing the IgE-CAI reaction were lower in EoDTR mice than in WT mice but were higher than those in BasoDTR mice (Fig. 3B). These results indicated that eosinophils are involved in the exacerbation of ear swelling, rather than in the induction of inflammation, in IgE-CAI.

**Figure 7:**
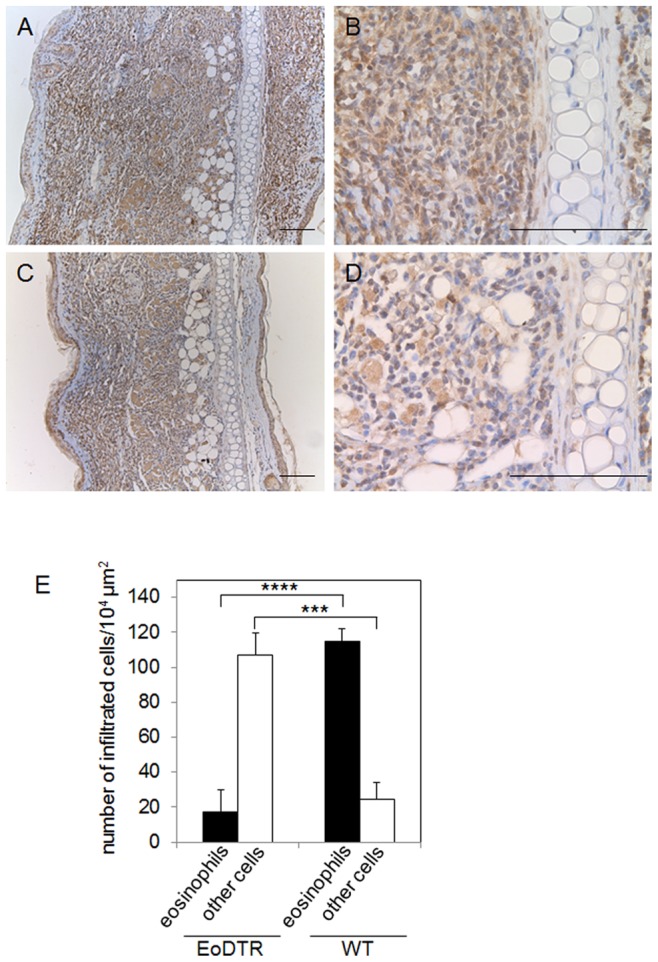
Histopathological analysis of the ear skin in chronic-phase swelling in IgE-CAI in EoDTR mice. WT (A and B) and EoDTR (C and D) mice were treated once i.p. with 5 µg/kg DT at 2 days before and were injected i.v. with 300 µg TNP-specific IgE at 1 day before challenge with 10 µg TNP-OVA. Ear specimens prepared 4 days after antigen challenge were incubated with the CCR3-specific antibody, stained with DAB (brown), and counterstained with hematoxylin (blue). Scale bars = 50 µm. Data are representative of 6 independent experiments. (E) The number of eosinophils and other cells in a 100-µm square was counted. Data have been expressed in terms of mean ± SD and are representative of 3 repeated experiments. The *p* values of DT-treated EoDTR mice versus DT-treated WT mice are indicated. ***p<0.005, **p<0.001. Statistical analysis was performed using the Student’s *t* test.

We performed histopathological comparison of the ear skin in the chronic phase of swelling in IgE-CAI. The ear specimens of WT and EoDTR mice at day 4 were stained with a CCR3-specific antibody. When 5 µg/kg of DT was administered 2 days before antigen challenge, WT mice exhibited remarkable infiltration of proinflammatory cells, including eosinophils (stained brown), at the site of swelling on day 4 (Fig. 7A and 7B). The histological features of WT and EoDTR mice without DT treatment were almost identical to those of WT mice with DT treatment (data not shown). In contrast, significant reduction in eosinophil infiltration into the swelling site was observed in DT-administered EoDTR mice (Fig. 7C and 7D). We counted the number of eosinophils and other cells in a 100-µm square. Although we found no difference in the total cell number per unit area between EoDTR and WT mice, >80% of the infiltrated cells were CCR3-positive eosinophils in WT mice, and the proportion of eosinophils had decreased to ∼15% in EoDTR mice (Fig. 7E). These results confirm that eosinophils are the principal effectors in the chronic phase of IgE-CAI, which is characterized by ear swelling.

## Discussion

In this study, we generated the conditional-deletion systems–BasoDTR and EoDTR mice–with the TRECK method by using the *CD203c* and *EPO* promoters, respectively. Using BasoDTR and EoDTR mice allowed us to clearly distinguish the *in vivo* functions of basophils and eosinophils in IgE-CAI. Basophils and eosinophils play important roles in various host defense mechanisms against bacteria, fungi, and parasites [35], [36], [37], [38], [39]. However, these cells also have harmful effects in allergic disorders such as atopic dermatitis and asthma. Basophils play an essential role in the development of the delayed-onset ear swelling reaction that is defined as IgE-CAI and is accompanied by massive eosinophil infiltration. It is believed that activated basophils secrete cytokines or chemokines to recruit and activate eosinophils [21]. The effector functions of eosinophils appear to be derived primarily from the release of lipid mediators and proteins, including cytokines and granule proteins. Eosinophil degranulation results in the release of several cytotoxic cationic granule proteins. Furthermore, the release of cytokines by eosinophils and other cells involved in inflammation amplifies and regulates localized immune responses [6].

A basophil-specific promoter should be used to specifically kill basophils by the TRECK method. Although a previous study revealed that CD203c is expressed not only in basophils but also in mast cells and their progenitors in humans [33], the expression specificity in the mouse is unknown. Therefore, we examined whether CD203c was expressed on mouse mast cells by using RT-PCR. *CD203c* mRNA was expressed in bone marrow-derived basophils but not in mast cells in peritoneal exudate cells from the mice (Fig. S1A). Thus, i.p. administration of DT to BasoDTR mice caused basophil ablation in the peripheral blood and spleen without affecting other cell types, including mast cells (Fig. 2 and Table 1). Moreover, DT-treated BasoDTR mice exhibited immediate-phase ear swelling (Fig. 3A) and IgE-mediated systemic anaphylaxis (Fig. 3B) as seen in WT mice, indicating that the mast cells of BasoDTR mice retained their normal functions.

Several groups have established mouse models with depleted basophils or eosinophils in order to study the critical roles of these cells in allergic reactions *in vivo*. One such model of basophil-deleted mice is constitutive basophil ablation (*Mcpt8Cre* mice) generated by the BAC-mediated transgenesis of *Cre* driven by the *Mcpt8* promoter, and another is DT-induced basophil ablation (*Mcpt8^DTR^* mice) generated by the transgenesis of the *hDTR* gene driven by the *Mcpt8* promoter. *Mcpt8Cre* mice exhibit a constitutive lack of basophils [27]. Studies using *Mcpt8Cre* mice have demonstrated that basophils function in protective immunity against helminthes and orchestrate chronic allergic inflammation, whereas primary Th2 cell responses operate efficiently in the absence of basophils [27]. In contrast, selective ablation of basophils occurred in *Mcpt8^DTR^* mice on DT administration. DT-mediated basophil ablation in *Mcpt8^DTR^* mice has been shown to abolish acquired tick resistance [26]. Moreover, a DT-based conditional basophil ablation mouse model, named Bas-TRECK, was generated using the *IL-4* promoter, 5′ enhancer, and proximal 3′ enhancer [28]. DT administration in Bas-TRECK mice resulted in basophil deletion both in the bone marrow and in peripheral blood. It was found that basophil deletion had no effect on the effector phase of allergic airway hyperresponsiveness or on IgE production induced by systemic OVA immunization.

The peripheral blood and spleens of the BasoDTR mice generated in this study were depleted of basophils after treatment with a dose of DT similar to that used in *Mcpt8^DTR^* and Bas-TRECK mice. Although basophils were depleted in both the peripheral blood and the bone marrow of *Mcpt8^DTR^* and Bas-TRECK mice [26], [28], bone marrow basophils were not depleted in our BasoDTR mice (Fig. 2). This result suggests that peripheral basophils are sufficient for the development of IgE-CAI. Therefore, this study offers a new line of evidence regarding the role of basophils in IgE-CAI, as bone marrow basophils appear to have no effect on IgE-CAI. We anticipate that the combination of these mouse models will provide new knowledge about basophil maturation. For example, using our mice, the cellular dynamics of basophils can be measured; depletion percentage of IgE-CAI can be measured through kinetic analysis during a time-course after DT administration. In contrast to the effect of DT administration on the basophil population in our BasoDTR mice, DT administration increased the basophil levels in the peripheral blood of WT mice, most likely as a consequence of the immunological response against DT. DT administration in BasoDTR mice completely suppressed ear swelling in IgE-CAI (Fig. 3A). Moreover, DT treatment in BasoDTR mice attenuated the drop in body temperature observed in systemic anaphylaxis elicited by i.v. injection of mAb against FcγRII/III in a dose-dependent manner (Fig. 4A). These results indicate that basophil functions were eliminated in BasoDTR mice by DT administration *in vivo* and that function is maintained only by peripheral basophils.

Unlike basophil-depleted mice, the EoDTR mice generated in this study represent the first model for conditional eosinophil ablation *in vivo*. The i.p. administration of DT to EoDTR mice resulted in eosinophil ablation in the peripheral blood and spleen, whereas the eosinophil population slightly increased in the bone marrow in these mice as well as in WT mice (Fig. 5).

We analyzed ear swelling in IgE-CAI in EoDTR mice. The ear swelling was remarkably ameliorated whether DT was administered before, at the same time as, or after the antigen challenge (Fig. 6B, C and D). When DT was administered at the same time as the antigen challenge, the ear swelling elicited was equal to that elicited in WT mice on days 1 and 2, was attenuated to half the level of that seen in WT mice on days 3 and 4, and returned to the original level after day 5. When DT was administered on day 2, ear swelling reduced from day 3 to day 6. Thus, although the eosinophil turnover was rapid, it appears that 1 dose of DT effected the ablation of eosinophil function for a minimum of 4 days. Notably, ear swelling was attenuated even when EoDTR mice were treated with DT after the start of the IgE-CAI response (Fig. 6D). This result suggests that eosinophil is a major aggravation factor in IgE-CAI and is therefore appropriate as a target for the cure of certain kinds of chronic allergic inflammation.

We used antibody arrays to semiquantitatively measure the cytokines and chemokines present in the ears 4 days after antigen injection in IgE-CAI. DT-administered BasoDTR mice exhibited lower concentrations of not only Th2 cytokines, but also Th1 cytokines and chemokines, than did WT and EoDTR mice (Fig. 3B). Ear swelling in IgE-CAI is primarily controlled by basophils and decreases upon infiltration of proinflammatory cells such as eosinophils and neutrophils [21]. Therefore, the expression of the cytokines and chemokines relevant to IgE-CAI were suppressed altogether in DT-administered BasoDTR mice. Furthermore, eotaxin-2, in particular, was expressed at higher levels than other chemokines in WT and EoDTR mice but its expression was diminished in BasoDTR mice. These results suggest that eotaxin-2 is a principal chemokine that attracts proinflammatory cells, leading to chronic allergic inflammation.

In conclusion, we generated novel Tg mouse models expressing hDTR exclusively on basophils and eosinophils. We confirmed that these Tg models showed improved allergic responses upon the depletion of basophils and eosinophils following DT administration. The combination of BasoDTR and EoDTR mouse models provides a new approach for clarifying complicated mechanisms of allergic inflammation such as those seen in atopic dermatitis and asthma.

## Supporting Information

Figure S1 **RT-PCR analysis of hDTR, CD203c, and EPO in WT, BasoDTR, and EoDTR mice.** Basophils and eosinophils were purified from the bone marrow and the spleen of WT, BaDTR, and EoDTR mice, and mast cells were purified from the peritoneal exudate cells of WT and BaDTR mice by using a positive selection of the MACS system with FITC-conjugated anti-IgE mAb, FITC-conjugated anti-CCR3 mAb, PE-conjugated anti-c-kit mAb, and anti-FITC or anti-PE microbeads. The expression of hDTR, CD203c, and EPO mRNA was analyzed by RT-PCR. PCR was performed on cDNA from the indicated cells using the following primer sets: 5′-TTA TCC TCC AAG CCA CAA GCA CTG-3′ and 5′-AGA CAG ACA GAT GAC AGC ACC ACA G-3′ for hDTR, 5′-TTC AGG AGC AAA GGG AGT TC-3′ and 5′-TGG GAG GAA GAG ATG ATG TG-3′ for CD203c, 5′-GCG GCT CCG TAA TAG GAC CAA C-3′ and 5′-GGA TAG GGT CGA TGC CAC CTT C-3′ for EPO, and 5′-AGG CCG GTG CTG AGT ATG TC-3′ and 5′-TGC CTG CTT CAC CAC CTT CT-3′ for GAPDH. Ba, BasoDTR; Eo, EoDTR; B, bone marrow; S, spleen; P, peritoneal exudate cells; GAPDH, glyceraldehyde-3-phosphate dehydrogenase.
(TIF)
