# Supplementary figures and images for: Novel Basophil- or Eosinophil-Depleted Mouse Models for Functional Analyses of Allergic Inflammation
Source: PLoS One. 2013 Apr 8;8(4):e60958. doi: 10.1371/journal.pone.0060958 (PMC3620047; doi:10.1371/journal.pone.0060958)

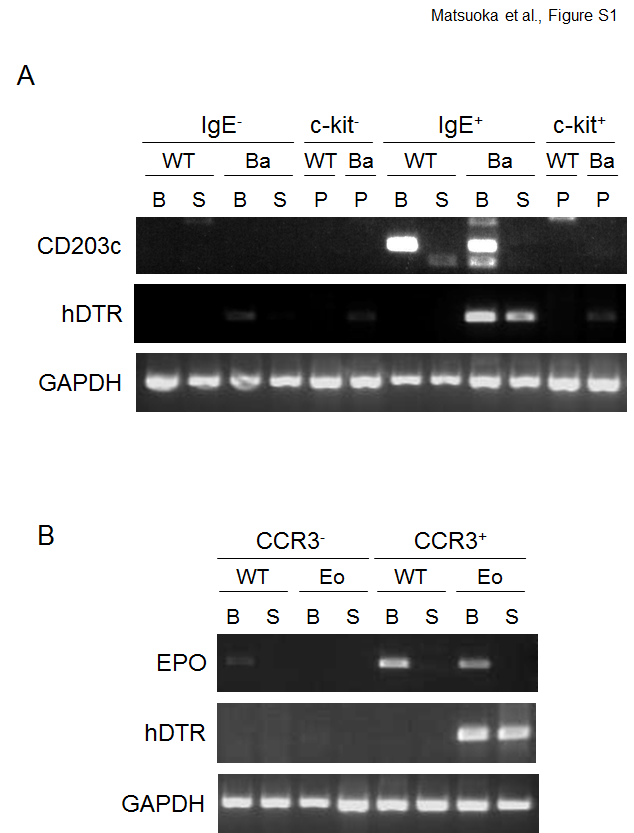

Supplement: Figure S1 — RT-PCR analysis of hDTR, CD203c, and EPO in WT, BasoDTR, and EoDTR mice. Basophils and eosinophils were purified from the bone marrow and the spleen of WT, BaDTR, and EoDTR mice, and mast cells were purified from the peritoneal exudate cells of WT and BaDTR mice by using a positive selection of the MACS system with FITC-conjugated anti-IgE mAb, FITC-conjugated anti-CCR3 mAb, PE-conjugated anti-c-kit mAb, and anti-FITC or anti-PE microbeads. The expression of hDTR, CD203c, and EPO mRNA was analyzed by RT-PCR. PCR was performed on cDNA from the indicated cells using the following primer sets: 5′-TTA TCC TCC AAG CCA CAA GCA CTG-3′ and 5′-AGA CAG ACA GAT GAC AGC ACC ACA G-3′ for hDTR, 5′-TTC AGG AGC AAA GGG AGT TC-3′ and 5′-TGG GAG GAA GAG ATG ATG TG-3′ for CD203c, 5′-GCG GCT CCG TAA TAG GAC CAA C-3′ and 5′-GGA TAG GGT CGA TGC CAC CTT C-3′ for EPO, and 5′-AGG CCG GTG CTG AGT ATG TC-3′ and 5′-TGC CTG CTT CAC CAC CTT CT-3′ for GAPDH. Ba, BasoDTR; Eo, EoDTR; B, bone marrow; S, spleen; P, peritoneal exudate cells; GAPDH, glyceraldehyde-3-phosphate dehydrogenase. (TIF) [file pone.0060958.s001.tif]
